# Supplementary material for: Impact of the Novel CoronaviruS (COVID-19) on Frontline PharmacIsts Roles and ServicEs: INSPIRE Worldwide Survey
Source: Pharmacy (Basel). 2023 Mar 29;11(2):66. doi: 10.3390/pharmacy11020066 (PMC10142295; doi:10.3390/pharmacy11020066)
Supplement: Supplementary file 1 [file pharmacy-11-00066-s001.zip › Supplement - survey.pdf]

Impact of the Novel CoronaviruS (COVID-19) on Frontline PharmacIsts Roles and ServicEs: INSPIRE Worldwide

Please complete the survey below.

Thank you!

Impact of the Novel CoronaviruS (COVID-19) on Frontline PharmacIsts Roles and ServicEs: INSPIRE Worldwide

Participant Information Sheet

(pro00108598)

Principal Investigator:

Dr. Yazid Al Hamarneh

Dept. of Medicine, University of Alberta

Yazid.Alhamarneh@ualberta.ca

Co-Investigators:

Dr. Dillon Lee

Resident Pharmacist and Student Researcher

dillon1@ualberta.ca

Dr. Kaitlyn Watson

Dept. of Medicine, University of Alberta

kewatson@ualberta.ca

Please refer to this information sheet for details regarding your rights while participating in the research study. Information listed in this letter applies to all aspects of your research participation. Print off the information sheet if you want to have a copy for your records.

Background: COVID-19 is continuing to plague the global community and impacts our healthcare system. Pharmacists have a key role to play in responding to the current pandemic and relieving pressure on the health service. We want to document and acknowledge the roles and services have been performing during the pandemic and identify the unique challenges being faced by pharmacists and the pharmacy profession.

Purpose: The purpose of this study is to identify the impact of COVID-19 on pharmacy practice worldwide.

Participants: The target audience for the survey is any pharmacist who has been working during the COVID-19 pandemic. This includes pharmacists who provide direct patient care or work in academic, government, COVID-19 task groups, and/or pharmacists in training (students, interns, residents).

We want to know what roles and services you have performed during COVID and the challenges you and your pharmacy practice have faced. There are over 2 million pharmacists practicing worldwide, we are ambitiously aiming for 5% of this population to complete the survey and responses from each country that has a pharmacy profession.

This survey will take approximately 5 minutes to complete.

Possible Benefits: There are no direct benefits to your participation in this study. However, you will be helping us document the valuable contributions that pharmacists have made to the pandemic.

Possible Risks/voluntary participation: There are no risks beyond the day-today for individuals participating in this survey.

Voluntary: Participation is optional, and you may withdraw from the study at any time. However, as we do not collect any personal information from you once the survey has been submitted, we are unable to remove your response.

Confidentiality: All information collected from you will be confidential and no personal identifiable information will be collected. As per our ethics policy, the University of Alberta requires us to keep data from the study for five years.

Reimbursement of Expenses: You will not be paid for participating in this study.

Contact Names and Telephone Numbers The plan for this study has been reviewed by a Research Ethics Board at the University of Alberta. If you have any questions regarding your rights as a research participant or how the research is being conducted you may contact the Research Ethics Office at 780-492-2615

---

1. Do you provide informed consent to participate in the research study?

○ Yes ○ No

Thank you for participating. Have a great day!

---

2. What is your practice setting during the COVID-19 Pandemic? Select all that apply.

- ☐ Community Pharmacy- Franchise or Chain (e.g., Shoppers Drug Mart, Boots)
- ☐ Community Pharmacy- Independent
- ☐ Team-based Community Practice (e.g., primary care network, family health team)
- ☐ Acute Care/Inpatient Care (e.g., hospital)
- ☐ Ambulatory/Outpatient Clinic (e.g., specialty clinics)
- ☐ Continuing or long-Term Care
- ☐ Academia (e.g., university, research, teaching)
- ☐ Government (e.g., Policy development)
- ☐ Non-government organization (e.g., Médecins Sans Frontières, Pharmaciens Sans Frontières)
- ☐ Regulatory organization (e.g., licensure, college of pharmacy)
- ☐ Others

If other, please specify:

____

---

3. In which country have you worked during COVID-19 pandemic (Please respond in English (e.g., Canada, India, United States, Spain, etc.))?

____

---

4. Please indicate if you have undertaken any of the below roles during any stage of the COVID-19 pandemic.
COVID-19 Roles

	Yes	No	Not applicable to my practice setting	Not applicable to my country
Addressing misinformation on COVID-19 treatments and vaccination to other healthcare providers (e.g., in-service, seminar, journal club etc.)	○	○	○	○

Addressing misinformation on COVID-19 treatments and vaccination to patients (e.g., counselling, posters, campaigns, etc.)	○	○	○	○
Allaying patients fears and anxiety about COVID-19	○	○	○	○
Administering the COVID-19 vaccine	○	○	○	○
Educating the public on reducing the spread of COVID-19 (e.g., handwashing, physical/social-distancing)	○	○	○	○
Participating on COVID-19 taskforce	○	○	○	○
Contributing to/establishing a field hospital for COVID-19	○	○	○	○
Working in a field hospital for COVID-19	○	○	○	○
Providing PPE supplies to patients (e.g., face masks)	○	○	○	○
Guiding policy development on COVID-19	○	○	○	○
Coordinating clinical trial management specific to COVID-19 treatments	○	○	○	○
Advocating for COVID-19 public health messaging (e.g., radio, television, community outreach)	○	○	○	○
Reporting domestic violence	○	○	○	○
Providing drive-thru pharmacy services (e.g., anticoagulant clinic, INR testing, medication dispensing, COVID-19 testing)	○	○	○	○
Providing Psychological First Aid (e.g., identifying patients at risk of mental health crises)	○	○	○	○
Providing telehealth or tele-pharmacy consults	○	○	○	○
Compounding hand sanitizers	○	○	○	○

Extension of Pharmacists' Everyday Roles

	Yes	No	Not applicable to my practice setting	Not applicable to my country
Rationing medicine supplies	◯	◯	◯	◯
Delivering medications to patients at home	◯	◯	◯	◯
Renewing/Extending prescriptions	◯	◯	◯	◯
Prescribing emergency supply refills	◯	◯	◯	◯
Administering influenza and other vaccines	◯	◯	◯	◯
Treating ambulatory conditions (e.g., mild constipation, mild pain)	◯	◯	◯	◯
Managing and/or monitoring patients' chronic diseases	◯	◯	◯	◯
Responding to drug information requests	◯	◯	◯	◯
Performing medication reviews	◯	◯	◯	◯
Others	◯	◯	◯	◯

If other, please specify: ______________________________

5. Did you engage with the local disaster and public health agencies to coordinate your pharmacy's response to COVID-19? ◯ Yes ◯ No

6. Were you consulted about COVID-19 in terms of pharmacist roles/expectations for pharmacy services? ◯ Yes ◯ No

If yes, by whom? (select all that apply)

- ☐ Government
- ☐ Public health services
- ☐ Local disaster response organizations
- ☐ Pharmacy organizations
- ☐ Administration teams in hospitals
- ☐ Corporate (e.g., Pharmacy chain, franchise)
- ☐ Others

If other, please specify: ______________________________

7. Which of the following challenges impacted you or your practice during any stage of the COVID-19 pandemic? Select all that apply.

- ☐ Lack of personal-protective equipment (e.g., face masks, gloves)
- ☐ Decreased supply of medications (e.g., medication shortage)
- ☐ General supply shortage (e.g., hand sanitizer)
- ☐ Inadequate staffing
- ☐ Lack of time for clinical counseling (e.g., care plan/medication management activities)
- ☐ HR-related issues (e.g., vacation time/ paid time off, sick/personal leave, scheduling flexibility, threat of repercussions for missing work, hazard pay)
- ☐ Inadequate time for breaks/meals
- ☐ Lack of access to COVID-19 testing (e.g., not supply issues, pharmacists on list of healthcare priority to receive priority vaccines)
- ☐ Lack of access to COVID-19 vaccines (e.g., not supply issues, pharmacists on list of healthcare priority to receive priority vaccines)
- ☐ Prescription surge (e.g., patient stockpiling)
- ☐ Unfair patient expectations (e.g., feeling frustrated with delay in services, abuse)
- ☐ Insurance issues (e.g., copay)
- ☐ Not feeling confident about delivering expanded scope of practice services during COVID-19 (e.g., extending opioids for continuity of care)
- ☐ Concern for safety (e.g., self and others)
- ☐ Increased stress level
- ☐ Time required to develop safety plans for COVID-19
- ☐ Extra cost associated with following the public health regulations (e.g., installing plexiglass, additional signage)
- ☐ Loss of business (e.g., reduced foot traffic, social distancing measures)
- ☐ Childcare (e.g., homeschooling)
- ☐ Correcting misinformation with patients (e.g., use of hydroxychloroquine to treat COVID-19 infection)
- ☐ Feeling unsure of pharmacy's role or responsibilities

---

8. How did you hear about this survey?

- ☐ Social media (e.g., Twitter, Facebook, LinkedIn, etc.)
- ☐ National/State pharmacy organization
- ☐ International pharmacy organization
- ☐ Other

If other, please specify: ______________________________

---

9. Do you have any other comments you'd like to add about pharmacists and COVID-19? ______________________________
