# Supplementary material for: Impact of the Novel CoronaviruS (COVID-19) on Frontline PharmacIsts Roles and ServicEs: INSPIRE Worldwide Survey
Source: Pharmacy (Basel). 2023 Mar 29;11(2):66. doi: 10.3390/pharmacy11020066 (PMC10142295; doi:10.3390/pharmacy11020066)
Supplement: Supplementary file 1 [file pharmacy-11-00066-s001.zip › Supplement table S1.pdf]

Table S1. Descriptive analysis of INSPIRE worldwide by WHO Region (N = 505)								
**Variable**		**African Region**	**Eastern Mediterranean Region**	**European Region**	**Missing**	**Region of the Americas**	**Western Pacific**	**Total**
		**N = 2**	**N =79**	**N = 12**	**N = 14**	**N = 357**	**N = 41**	**N = 505**
		N (%)	N (%)	N (%)	N (%)	N (%)	N (%)	N (%)
Addressing misinformation on COVID-19 treatments and vaccination to other healthcare providers					n =12			n= 503
	1. Yes	1(50.00)	38(48.10)	5(41.67)	8(66.67)	151(42.30)	21(51.22)	224(44.53)
	2. No	0	26(32.91)	5(41.67)	3(25.00)	159(44.54)	14(34.15)	207(41.15)
	3. Not applicable to my practice setting	1(50.00)	13(16.46)	2(16.67)	0	47(13.17)	6(14.63)	69(13.72)
	4. Not applicable to my country	0	2(2.53)	0	1(8.33)	0	0	3(0.60)
Addressing misinformation on COVID-19 treatments and vaccination to patients					n = 11			n= 502
	1. Yes	1(50.00)	52(65.82)	9(75.00)	8(11)	291(81.51)	36(87.80)	397(79.08)
	2. No	0	20(25.32)	2(16.67)	3(27.27)	45(12.61)	4(9.76)	74(14.74)
	3. Not applicable to my practice setting	0	7(8.86)	1(8.33)	0	21(5.88)	1(2.44)	30(5.98)
	4. Not applicable to my country	1(50.00)	0	0	0	0	0	1(0.20)
Allaying patients fears and anxiety about COVID-19					n =11			n= 499
	1. Yes	1(50.00)	61(80.26)	9(75.00)	8(72.73)	298(83.47)	36(87.80)	413(82.77)
	2. No	0	12(15.79)	1(8.33)	3(27.27)	45(12.61)	3(7.32)	64(12.83)
	3. Not applicable to my practice setting	0	2(2.63)	2(16.67)	0	14(3.92)	2(4.88)	20(4.01)
	4. Not applicable to my country	1(50.00)	1(1.32)	0	0	0	0	2(0.40)
Administering the COVID-19 vaccine					n =11	n = 356		n= 501
	1. Yes	0	26(32.91)	3(25.00)	9(81.82)	183(51.40)	4(9.76)	225(44.91)
	2. No	0	39(49.37)	4(33.33)	2(18.18)	126(35.39)	26(63.41)	197(39.32)
	3. Not applicable to my practice setting	0	9(11.39)	4(33.33)	0	47(13.20)	6(14.63)	66(13.17)
	4. Not applicable to my country	2(100.00)	5(6.33)	1(8.33)	0	0	5(12.20)	13(2.59)
Educating the public on reducing the spread of COVID-19					n =12			n = 503
	1. Yes	2(100.00)	70(88.61)	9(75.00)	11(91.67)	280(78.43)	37(90.24)	409(81.31)
	2. No	0	6(7.59)	0	1(8.33)	58(16.25)	1(2.44)	66(13.12)
	3. Not applicable to my practice setting	0	2(2.53)	3(25.00)	0	19(5.32)	3(7.32)	27(5.37)
	4. Not applicable to my country	0	1(1.27)	0	0	0	0	1(0.20)
Participating on COVID-19 taskforce			n =78		n =10	n = 355		n = 498
	1. Yes	0	30(38.46)	3(24.00)	6(60.00)	74(20.85)	14(34.15)	127(25.50)
	2. No	2(100.00)	35(44.87)	8(66.67)	4(40.00)	235(66.20)	15(36.59)	299(60.04)
	3. Not applicable to my practice setting	0	11(14.1)	1(8.33)	0	46(12.96)	11(26.83)	69(13.86)
	4. Not applicable to my country	0	2(2.56)	0	0	0	1(2.44)	3(0.60)
Contributing to/establishing a field hospital for COVID-19			n = 78		n = 11	n =355		n=499
	1. Yes	0	17(21.79)	2(16.67)	4(36.36)	29(8.17)	3(7.32)	55(11.02)
	2. No	0	49(62.82)	9(75.00)	6(54.55)	234(65.92)	22(53.66)	320(64.13)
	3. Not applicable to my practice setting	0	12(15.38)	1(8.33)	1(9.09)	91(25.63)	12(29.27)	117(23.45)
	4. Not applicable to my country	2(100.00)	0	0	0	1(0.28)	4(9.76)	7(1.40)

Working in a field hospital for COVID-19					n =11	n =355		n=500
	1. Yes	0	11(13.92)	1(8.33)	4(36.36)	8(2.25)	4(9.76)	28(5.60)
	2. No	2(100.00)	54(68.35)	10(83.33)	5(45.45)	251(70.70)	23(56.10)	345(69.00)
	3. Not applicable to my practice setting	0	13(16.46)	1(8.33)	2(18.18)	94(26.48)	9(21.95)	119(23.80)
	4. Not applicable to my country	0	1(1.27)	0	0	2(0.56)	5(12.20)	8(1.60)
Providing PPE supplies to patients (e.g., face masks)			n =77		n =12	n =356		n= 500
	1. Yes	2(100.00)	50(64.94)	5(41.67)	9(75.00)	159(44.66)	33(80.49)	258(51.60)
	2. No	0	20(25.97)	6(50.00)	3(25.00)	158(44.38)	5(12.20)	192(38.40)
	3. Not applicable to my practice setting	0	6(7.79)	1(8.33)	0	39(10.96)	3(7.32)	49(9.80)
	4. Not applicable to my country	0	1(1.3)	0	0	0	0	1(0.20)
Guiding policy development on COVID-19		n = 2	n =79	n = 12	n =11	n = 355	n = 41	n=500
	1. Yes	0	34(43.04)	3(25.00)	6(54.55)	87(24.51)	9(21.95)	139(27.80)
	2. No	1(50.00)	33(41.77)	8(66.67)	4(36.36)	220(61.97)	18(43.90)	284(56.80)
	3. Not applicable to my practice setting	0	9(11.39)	1(8.33)	1(9.09)	48(13.52)	14(34.15)	73(14.60)
	4. Not applicable to my country	1(50.00)	3(3.8)	0	0	0	0	4(0.80)
Coordinating clinical trial management specific to COVID-19 treatments				n = 11	n =11	n =355		n=499
	1. Yes	0	18(22.78)	0	4(36.36)	26(7.32)	3(7.32)	51(10.22)
	2. No	0	50(63.29)	11(100.00)	6(54.55)	244(68.73)	23(56.10)	334(66.93)
	3. Not applicable to my practice setting	0	7(8.86)	0	1(9.09)	85(23.94)	14(34.15)	107(21.44)
	4. Not applicable to my country	2(100.00)	4(5.06)	0	0	0	1(2.44)	7(1.40)
Advocating for COVID-19 public health messaging			n =78		n =11	n =355		n= 499
	1. Yes	0	38(48.72)	3(25.00)	8(72.73)	67(18.87)	11(26.83)	127(25.45)
	2. No	1(50.00)	32(41.03)	8(66.67)	3(27.27)	229(64.51)	17(41.46)	290(58.12)
	3. Not applicable to my practice setting	0	7(8.97)	1(8.33)	0	59(16.62)	12(29.27)	79(15.83)
	4. Not applicable to my country	1(50.00)	1(1.28)	0	0	0	1(2.44)	3(0.60)
Reporting domestic violence					n =11	n = 355	n = 40	n= 499
	1. Yes	0	17(21.52)	1(8.33)	3(27.27)	20(5.63)	2(5.00)	43(8.62)
	2. No	1(50.00)	48(60.76)	10(83.33)	8(72.73)	279(78.59)	32(80.00)	378(75.75)
	3. Not applicable to my practice setting	0	11(13.92)	1(8.33)	0	55(15.49)	5(12.50)	72(14.43)
	4. Not applicable to my country	1(50.00)	3(3.8)	0	0	1(0.28)	1(2.50)	6(1.20)
Providing drive-thru pharmacy services					n =11	n =354		n= 499
	1. Yes	1(50.00)	28(35.44)	1(8.33)	7(63.64)	79(22.32)	6(14.63)	122(24.45)
	2. No	1(50.00)	37(46.84)	9(75.00)	4(36.36)	187(52.82)	21(51.22)	259(51.90)
	3. Not applicable to my practice setting	0	10(12.66)	2(16.67)	0	85(24.01)	10(24.39)	107(21.44)
	4. Not applicable to my country	0	4(5.06)	0	0	3(0.85)	4(9.76)	11(2.20)
Providing Psychological First Aid					n =11	n =355		n= 500
	1. Yes	1(50.00)	30(37.97)	1(8.33)	5(45.45)	61(17.18)	8(19.51)	106(21.20)
	2. No	0	40(50.63)	8(66.67)	6(54.55)	237(66.76)	24(58.54)	315(63.00)
	3. Not applicable to my practice setting	0	7(8.86)	3(25.00)	0	55(15.49)	7(17.07)	72(14.40)
	4. Not applicable to my country	1(50.00)	2(2.53)	0	0	2(0.56)	2(4.88)	7(1.40)

Providing telehealth or tele-pharmacy consults					n =12	n =355		n= 501
	1. Yes	0	43(54.43)	5(41.67)	7(58.33)	159(44.79)	12(29.27)	226(45.11)
	2. No	0	26(32.91)	5(41.67)	5(41.67)	150(42.25)	15(36.59)	201(40.12)
	3. Not applicable to my practice setting	0	6(7.59)	2(16.67)	0	46(12.96)	12(29.27)	66(13.17
	4. Not applicable to my country	2(100.00)	4(5.06)	0	0	0	2(4.88)	8(1.60)
Compounding hand sanitizers					n =11	n =355		n= 500
	1. Yes	2(100.00)	38(48.10)	5(41.67)	2(18.18)	26(7.32)	11(26.83)	84(16.80)
	2. No	0	28(35.44)	4(33.33)	9(81.82)	255(71.83)	22(53.66)	318(63.60)
	3. Not applicable to my practice setting	0	10(12.66)	3(25.00)	0	73(20.56)	7(17.07)	93(18.60)
	4. Not applicable to my country	0	3(3.8)	0	0	1(0.28)	1(2.44)	5(1.00)
Rationing medicine supplies			n = 78		n =11			n=501
	1. Yes	1(50.00)	53(67.95)	8(66.67)	7(63.64)	239(66.95)	33(80.49)	341(68.06)
	2. No	0	15(19.23)	3(25.00)	4(36.36)	80(22.41)	5(12.20)	107(21.36)
	3. Not applicable to my practice setting	0	9(11.54)	1(8.33)	0	38(10.64)	3(7.32)	51(10.18)
	4. Not applicable to my country	1(50.00)	1(1.28)	0	0	0	0	2(0.40)
Delivering medications to patients at home					n =11	n =356		n=501
	1. Yes	0	43(54.43)	5(41.67)	9(81.82)	186(52.25)	29(70.73)	272(54.29)
	2. No	1(50.00)	23(29.11)	4(33.33)	2(18.18)	93(26.12)	5(12.20)	128(25.55)
	3. Not applicable to my practice setting	0	11(13.92)	3(25.00)	0	77(21.63)	6(14.63)	97(19.36)
	4. Not applicable to my country	1(50.00)	2(2.53)	0	0	0	1(2.44)	4(0.80)
Renewing/Extending prescriptions					n = 12	n =356		n=502
	1. Yes	0	50(63.29)	7(58.33)	9(75.00)	207(58.15)	20(48.78)	293(58.37)
	2. No	1(50.00)	19(24.05)	1(8.33)	3(25.00)	73(20.51)	11(26.83)	108(21.51)
	3. Not applicable to my practice setting	0	10(12.66)	3(25.00)	0	75(21.07)	5(12.20)	93(18.53)
	4. Not applicable to my country	1(50.00)	0	1(8.33)	0	1(0.28)	5(12.20)	8(1.59)
Prescribing emergency supply refills					n =11	n =356		n=501
	1. Yes	2(100.00)	42(53.16)	5(41.67)	6(54.55)	173(48.60)	27(65.85)	255(50.90)
	2. No	0	23(29.11)	1(8.33)	5(45.45)	95(26.69)	6(14.63)	130(25.95)
	3. Not applicable to my practice setting	0	13(16.46)	5(41.67)	0	86(24.16)	6(14.63)	110(21.96)
	4. Not applicable to my country	0	1(1.27)	1(8.33)	0	2(0.56)	2(4.88)	6(1.20)
Administering influenza and other vaccines					n =11			n=502
	1. Yes	0	36(45.57)	4(33.33)	8(72.73)	216(60.50)	14(34.15)	278(55.38)
	2. No	0	29(36.71)	2(16.67)	3(27.27)	93(26.05)	16(39.02)	143(28.49)
	3. Not applicable to my practice setting	0	11(13.92)	4(33.33)	0	48(13.45)	5(12.20)	68(13.55)
	4. Not applicable to my country	2(100.00)	3(3.8)	2(16.67)	0	0	6(14.63)	13(2.59)
Treating ambulatory conditions					n =11	n =356		n= 501
	1. Yes	1(50.00)	58(73.42)	5(41.67)	9(81.82)	231(64.89)	26(63.41)	330(65.87)
	2. No	1(50.00)	12(15.19)	3(25.00)	2(18.18)	65(18.26)	9(21.95)	92(18.36)
	3. Not applicable to my practice setting	0	9(11.39)	4(33.33)	0	60(16.85)	5(12.2)	78(15.57)
	4. Not applicable to my country	0	0	0	0	0	1(2.44)	1(0.20)

Managing and/or monitoring patients' chronic diseases				n = 11	n = 12	n =356		n= 501
	1. Yes	1(50.00)	53(67.09)	6(54.55)	7(58.33)	267(75.00)	28(68.29)	362(72.26)
	2. No	1(50.00)	15(18.99)	2(18.18)	5(41.67)	50(14.04)	8(19.51)	81(16.17)
	3. Not applicable to my practice setting	0	11(13.92)	3(27.27)	0	39(10.96)	5(12.20)	58(11.58)
	4. Not applicable to my country	0	0	0	0	0	0	0
Responding to drug information requests					n =11		n =40	n= 501
	1. Yes	2(100.00)	68(86.08)	9(75.00)	10(90.91)	324(90.76)	35(87.50)	448(89.42)
	2. No	0	5(6.33)	2(16.67)	1(9.09)	21(5.88)	4(10.00)	33(6.59)
	3. Not applicable to my practice setting	0	6(7.59)	1(8.33)	0	12(3.36)	1(2.50)	20(3.99)
	4. Not applicable to my country	0	0	0	0	0	0	0
Performing medication reviews			n =78		n =10	n =355		n=498
	1. Yes	1(50.00)	58(74.36)	7(58.77)	8(80.00)	290(81.69)	27(65.85)	391(78.51)
	2. No	1(50.00)	12(15.38)	3(25.00)	2(20.00)	46(12.96)	10(24.39)	74(14.86)
	3. Not applicable to my practice setting	0	7(8.97)	2(16.67)	0	19(5.35)	4(9.76)	32(6.43)
	4. Not applicable to my country	0	1(1.28)	0	0	0	0	1(0.20)
